# The impact of social and psychological consequences of disease on judgments of disease severity: An experimental study

**DOI:** 10.1371/journal.pone.0195338

**Published:** 2018-04-17

**Authors:** Nicholas B. King, Sam Harper, Meredith Young, Sarah C. Berry, Kristin Voigt

**Affiliations:** 1 Biomedical Ethics Unit, McGill University, Montreal, Québec, Canada; 2 Department of Epidemiology, Biostatistics & Occupational Health, McGill University, Montreal, Québec, Canada; 3 Institute for Health and Social Policy, McGill University, Montreal, Québec, Canada; 4 Department of Medicine, McGill University, Montreal, Québec, Canada; 5 Centre for Medical Education, McGill University, Montreal, Québec, Canada; Scientific Institute of Public Health (WIV-ISP), BELGIUM

## Abstract

**Background:**

The Global Burden of Disease (GBD) project systematically assesses mortality, healthy life expectancy, and disability across 195 countries and territories, using the disability-adjusted life year (DALY). Disability weights in the DALY are based upon surveys that ask users to rate health states based on lay descriptions. We conducted an experimental study to examine whether the inclusion or removal of psychological, social, or familial implications from a health state description might affect individual judgments about disease severity, and thus relative disability weights.

**Methods:**

We designed a survey consisting of 36 paired descriptions in which information about plausible psychological, social, or familial implications of a health condition was either present or absent. Using a Web-based platform, we recruited 1,592 participants, who were assigned to one of two experimental groups, each of which were asked to assign a value to the health state description from 0 to 100 using a slider, with 0 as the “worst possible health” and 100 as the “best possible health.” We tested five hypotheses: (1) the inclusion of psychological, social, or familial consequences in health state descriptions will reduce the average rating of a health state; (2) the effect will be stronger for diseases with lower disability weights (i.e., less severe diseases); (3) the effect will vary across the type of additional information added to the health state description; (4) the impact of adding information on familial consequences will be stronger for female than male; (5) the effect of additional consequences on ratings of health state descriptions will not differ by levels of completed education and age.

**Results:**

On average, adding social, psychological, or familial consequences to the health state description lowered individual ratings of that description by 0.78 points. The impact of adding information had a stronger impact on ratings of the least severe conditions, reducing average ratings in this category by 1.67 points. Addition of information about child-rearing had the strongest impact, reducing average ratings by 2.09 points. We found little evidence that the effect of adding information on ratings of health descriptions varied by gender, education, or age.

**Conclusions:**

Including information about health states not directly related to major functional consequences or symptoms, particularly with respect to child-rearing and specifically for descriptions of less severe conditions, can lead to lower ratings of health. However, this impact was not consistent across all conditions or types of information, and was most pronounced for inclusion of information about child-rearing, and among the least severe conditions.

## Background

The Global Burden of Disease (GBD) project systematically assesses mortality, healthy life expectancy, and disability across 195 countries and territories, using the disability-adjusted life year (DALY), which incorporates mortality and morbidity into a single metric.[1]

After the DALY was first introduced in 1990, the GBD’s methods were criticized on the grounds that ‘health’ cannot—or should not—be separated from general welfare, which is shaped not only by an individual’s symptoms, but also by the interaction between those symptoms and the social environment.[2, 3] Individuals in different social or cultural milieus may experience the same symptom (e.g. reduced mobility or poor eyesight) differently, and symptoms or health conditions that in one context might pose few problems for social interactions might in another context be strongly stigmatized and thus profoundly disabling.[4, 5] Because these contexts vary between and within countries, and may also vary depending on an individual’s relative social position, the use of universal disability weights was critiqued as misguided.

Responding to these concerns, the 2010 GBD update sought to isolate the impact of health loss from the broader concept of welfare loss, thus reducing the possibility that differences in contextual factors might lead to systematic variations in health assessments across settings.[6, 7] In the 2010 GBD, respondents were asked to rate which of two hypothetical individuals is ‘healthier,’ on the basis of ‘brief lay descriptions that emphasised the major functional consequences and symptoms associated with each health state with simple, non-clinical vocabulary… that aimed to capture the most salient details for each health state, while ensuring consistency in wording across states and avoiding ambiguous terms.’[6]

GBD researchers argued that the use of parsimonious descriptions effectively isolated ‘health’ from welfare considerations, resulting in exceptionally consistent disability weights across countries and social groups. They thus concluded that

‘we did not observe evidence to support the hypothesis that comparative assessments of health at a global level are undermined by extensive cultural variation. On the contrary, we have reported strong evidence that many aspects of individuals’ assessments of health outcomes seem to reflect common values, affirming universal aspirations for averting negative health outcomes such as pain or depression and for enjoying high levels of functioning in domains of health such as mobility.’

[6]

While the GBD 2010 methodology was generally consistent, there were potentially important variations in the language used to describe health states. In particular, the descriptions for some health states included not only information about symptoms and functional consequences, but also consequences unrelated to health *per se*. This additional information included psychological consequences (e.g. anxiety about a diagnosis or recurring symptoms), implications for social interaction (e.g. a condition causes others to “stare and comment”), and/or implications for child-rearing (e.g. a condition makes it difficult to care for children). Moreover, the inclusion of psychological, social, or child-rearing (hereafter “familial”) implications was applied inconsistently across disease states: only some health state descriptions in which these implications are likely actually mentioned them. For example, psychological implications (anxiety) were included in the description of epilepsy, but not in the description of asthma:

**Epilepsy**: had sudden seizures in the past, but they have stopped now with medicines. The person has some drowsiness, difficulty concentrating and *some anxiety about future episodes*. [emphasis added]

**Asthma**: has wheezing, cough and shortness of breath more than twice a week, which causes difficulty with daily activities and sometimes wakes the person at night.

The inconsistent inclusion of non-health information raises two concerns. First, it calls into question the claim that ‘health’ was consistently isolated from welfare considerations in all health state descriptions. Second, if extra information affected evaluators’ ratings of the health states, then the inconsistent inclusion of this information may have influenced the relative disability weights of the health states. There is evidence that evaluative judgments may be subject to an “unpacking effect,” in which more detailed descriptions of a category or event facilitates the generation of evaluative evidence, which in turn produces more extreme evaluations of those categories or events, including health and suffering.[8]

We conducted an experimental study to examine whether the inclusion or removal of psychological, social, or familial implications from a health state description might affect individual judgments about disease severity, and thus relative disability weights.

## Methods

### Recruitment

We selected our study population using Crowdflower (http://www.crowdflower.com), a Web-based platform for recruiting and paying subjects to perform tasks. Crowdflower offers a wide selection of compensated tasks to a participant pool of over 1 million participants worldwide, and provides demographic information about its source population, which allowed us to examine the overall characteristics and representativeness of our participant pool. The use of an online platform for study recruitment and execution allowed for a larger and more representative sample than in-person convenience sampling.[9, 10] The Crowdflower system also allowed us to introduce safeguards into the administration of our survey, including specification of primary language; a minimum time allotment for the survey; rules to guard against participants completing the same survey multiple times (a “maximum judgments” option); selecting from a category of experienced and validated participants; and the collection of identifying information about participants (e.g. internet service provider (IP) addresses and worker identification (ID) numbers), which allowed us to exclude participants who may have attempted to take the surveys multiple times. Crowdflower offers three primary options for recruiting participants: 1) by level of trustworthiness/accuracy; 2) by country; 3) by primary language spoken. We entered our specifications into Crowdflower for these options (highest trustworthiness level, no specified country, English), and Crowdflower selected participants from their existing participant base.

Based on the findings of two pilot surveys of 50 participants each, we adopted the following strategy to exclude low-quality responses: upon completion of the surveys, we deleted (1) any surveys that were incomplete, on the grounds that they likely indicated that participants were not taking the survey seriously, and thus were unlikely to produce useful individual answers, with the exception of participants who did not complete the demographic information, since this content was optional; (2) any surveys in which participants answered any one of 5 dummy questions incorrectly; and (3) any survey that was completed in less than 4 minutes, a minimum time allotment determined by having a research assistant complete the survey in the pilot phase. In cases where we detected duplicate IP addresses or worker IDs, we retained the earliest results and deleted the others. Our surveys were taken by 3012 respondents; surveys were run sequentially within one day of each other. After excluding surveys that were incomplete (n = 205), surveys with at least one incorrect dummy question (n = 553), and surveys completed in less than 4 minutes (n = 662), we accepted surveys with n = 803 for version 1 and n = 789 for version 2. Results from the pilot surveys were not included in the final analysis.

We collected optional demographic information on the respondent’s age (continuous), gender (male, female), highest level of education (less than high school, high school diploma, university degree, graduate or professional degree), and race-ethnicity (using United States Census categories). Our sample was drawn from literate individuals with computer and internet access.

### Ethics approval and consent to participate

This study was approved by the McGill University Faculty of Medicine’s Research Ethics Office (IRB Study Number A09-B44-10B). All participants provided written consent for participation.

### Materials

We selected 36 different health state descriptions across the spectrum of disease severity from the GBD 2010. GBD health state descriptions were ranked by disability weight, then divided into low (disability weight <0.25), moderate (disability weight 0.24–0.5), and high (disability weight >0.5) severity category. We selected 12 descriptions from each category, choosing health state descriptions that were amenable to addition of social, psychological, or familial implications.

For each health state description, we designed a ‘paired’ description by either adding or removing information about plausible psychological, social, or familial implications of a health condition, based on the original health state descriptions published in [7]. We constructed two survey versions containing all 36 health state descriptions (S1 Text). The order of the descriptions was identical, but whether or not each description contained additional information varied—that is, for any given description, one survey contained the version with additional information, and the other survey contained the version without. Both surveys included descriptions with and without additional information in order to mimic the original GBD survey content. Health state descriptions contained only information about the symptoms of a health state, and did not name the health state.

**Fig 1:**
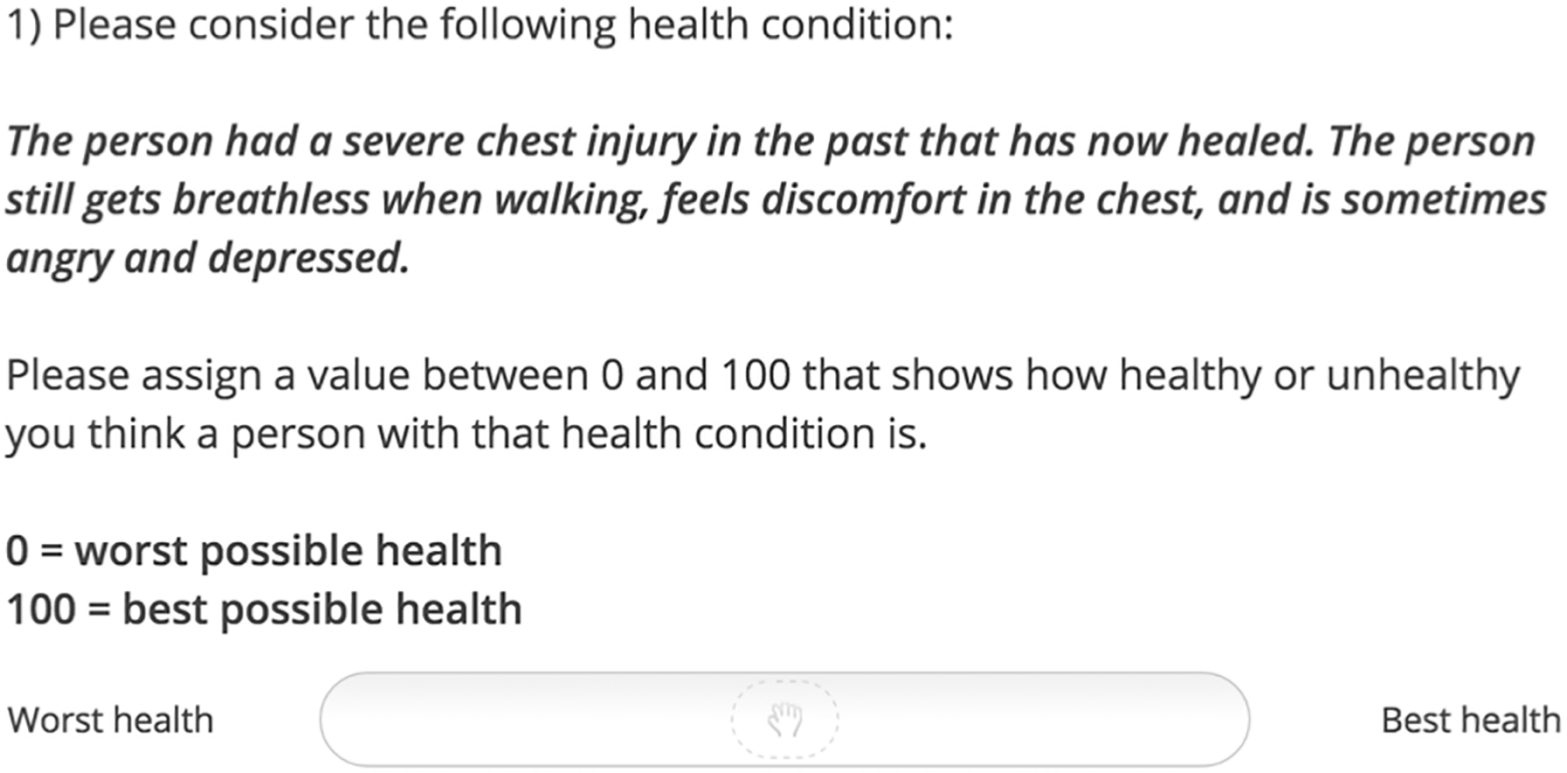
Sample question, including psychological consequences of health state.

In order to maximize variance across health state descriptions,[11] we used a modified visual analog scale (VAS) which allowed respondents to assign a precise numeric value to the health state description from 0 to 100 using a slider, with 0 as the “worst possible health” and 100 as the “best possible health” (Fig 1). As a check on the quality of the responses, we included 5 “dummy” questions throughout the survey that required the respondent to set the slider to a specific value (e.g., “Set the rating for this person at 78”) or included an unambiguous description of a 0 valuation (e.g. “The person is not breathing and has no pulse. The person is dead”).

### Statistical analysis

We tested five hypotheses: (1) the inclusion of psychological, social, or familial consequences in health state descriptions will reduce the average rating of a health state; (2) the effect will be stronger for diseases with lower disability weights (i.e., less severe diseases); (3) the effect will vary across the type of additional information added to the health state description; (4) the impact of adding information on familial will be stronger for females than males; (5) we also tested whether the effect of additional consequences on ratings of health state descriptions differed by levels of completed education and age.

We used linear regression models to estimate the impact of adding psychological, social, or family/familial information to each disease condition:

$$y_{i}=\alpha +x\beta +\varepsilon_{i}$$ (1)

where *y*_*i*_ is the health ranking of subject *i* and *x* is an indicator variable for whether the survey contained additional information. We used Eq (1) across all 36 conditions to estimate the average effect, and in subsequent models we also added demographic covariates and indicator variables for each question to estimate a conditional effect. For hypothesis (2) we extended Eq (1) to allow the effect to differ across levels of disability weight:

$$y_{i}=\alpha +x\beta +z\gamma +xz\delta +\varepsilon_{i}$$ (2)

where *y*_*i*_ and *x* are defined as in Eq (1), *z* is a continuous variable representing the disability weight as published in the original 2010 GBD survey [7] and *γ* is the coefficient corresponding to the independent effect of a one-unit change in disability weight, which varies from 0 (least disabling) to 1 (most disabling). A test of the coefficient δ = 0 provides evidence of a departure from additive effects of additional information and disability weight. We used a model similar to (2) to test hypotheses 3–5, all of which allowed the effect of adding information to vary by other characteristics. We used cluster robust standard errors to account for non-independence of responses among individuals—i.e., standard errors were clustered at the individual level.[12]

All of our analyses were pre-specified and conducted using Stata software, version 14 (Statacorp, College Station, TX). A copy of our pre-analysis plan is registered on the Experiments in Government and Political Science web portal (http://egap.org/registration/740).

## Results

Table 1 shows basic descriptive statistics and Pearson chi-square tests for independence for our two survey samples. Overall, the two surveys are balanced with respect to age, gender, race, education, and average survey completion time. There are minor differences for some categories. As a sensitivity analysis, we control for these demographic characteristics in regression models.

**Table 1:** Demographic characteristics of the study participants in survey versions 1 and 2.

	Survey version					
	Version 1	Version 1	Version 2	Version 2	Total	Total
	N	Col %	N	Col %	N	Col %
Age group						
14-24y	159	20	193	24.6	352	22.3
25-34y	341	42.9	324	41.2	665	42.1
35-44y	177	22.3	179	22.8	356	22.5
45+	117	14.7	90	11.5	207	13.1
Total	794	100	786	100	1,580	100
Pearson chi2(3) = 7.21, p = 0.065						
Gender						
Male	546	69	585	74.7	1,131	71.9
Female	245	31	198	25.3	443	28.1
Total	791	100	783	100	1,574	100
Pearson chi2(1) = 6.29, p = 0.012						
Race						
White	593	74.4	564	71.8	1,157	73.1
Indian	57	7.2	72	9.2	129	8.2
Asian	47	5.9	64	8.2	111	7
Other	100	12.5	85	10.8	185	11.7
Total	797	100	785	100	1,582	100
Pearson chi2(3) = 6.20, p = 0.102						
Highest education completed						
High school or less	277	35.6	265	34.3	542	35
University degree	317	40.8	345	44.7	662	42.7
Graduate school or higher	183	23.6	162	21	345	22.3
Total	777	100	772	100	1,549	100
Pearson chi2(2) = 2.71, p = 0.258						
Survey time to completion						
<10 mins	217	27	180	22.8	397	24.9
10–19 mins	503	62.6	444	56.3	947	59.5
20+ mins	83	10.3	165	20.9	248	15.6
Total	803	100	789	100	1,592	100
Pearson chi2(2) = 34.12, p < 0.001						
N	803		789		1,592	

Table 2 shows the health state name (the health state name was omitted from the actual surveys), average rating for each version of the survey, which version of the survey contained additional information, and the crude effect of adding information. Questions 15, 21, and 33 were dummy questions and were thus omitted from our analysis. Average ratings were lowest (~15) for conditions such as paralysis or severe cognitive and motor impairment, and highest (~70) for impotence. Adding information increased ratings for some questions and decreased ratings for others.

**Table 2:** Question description, mean ratings for each survey, and crude difference between questions with and without additional information.

				95% CI	
GBD Description	Rating with added info:	Rating without added info:	Difference	Lower	Upper
Spinal cord lesion at neck: treated	14.6	14.3	0.4	-1.1	1.8
Spinal cord lesion at neck level: untreated	15.4	14.6	0.8	-0.5	2.1
Motor plus cognitive impairments: severe	17.3	16.0	1.3	-0.1	2.8
Stroke: severe plus cognition problems	16.7	18.3	-1.6	-3.1	-0.2
Stroke: severe	19.1	20.6	-1.5	-3.1	0.1
Spinal cord lesion below neck: untreated	24.1	24.6	-0.5	-2.0	1.0
Dementia: severe	28.3	29.6	-1.3	-3.2	0.6
Multiple sclerosis: severe	29.9	30.4	-0.5	-2.1	1.1
End-stage renal disease: on dialysis	33.3	28.1	5.2	3.5	6.9
Parkinson’s disease: severe	32.5	33.4	-0.9	-2.5	0.7
Traumatic brain injury: long-term, severe	32.5	33.9	-1.4	-3.1	0.4
Multiple sclerosis: moderate	33.5	34.3	-0.8	-2.4	0.8
Musculoskeletal problems: generalised, severe	34.5	35.7	-1.2	-2.9	0.5
Rectovaginal fistula	34.4	35.9	-1.5	-3.4	0.4
Terminal phase: with medication	36.3	34.0	2.3	0.4	4.2
Musculoskeletal problems: generalised, moderate	35.7	34.8	0.8	-0.8	2.5
Intellectual disability: profound	35.6	38.4	-2.8	-4.9	-0.7
Motor plus cognitive impairments: moderate	37.5	40.1	-2.6	-4.4	-0.8
Heroin and other opioid dependence	39.1	40.6	-1.5	-3.6	0.5
AIDS cases: not receiving antiretroviral treatment	43.1	41.2	1.9	0.1	3.7
Distance vision: severe impairment	42.4	42.6	-0.2	-2.1	1.8
Stoma	43.8	41.7	2.2	0.2	4.1
Burns: short term	45.5	40.8	4.7	2.8	6.6
Bipolar disorder: manic episode	42.5	45.2	-2.7	-4.7	-0.7
Traumatic brain injury: long-term, moderate	39.6	48.7	-9.1	-10.9	-7.2
Diarrhoea: severe	43.9	45.7	-1.8	-3.8	0.2
Schizophrenia, residual state	42.3	47.6	-5.2	-7.1	-3.4
Vesicovaginal fistula	44.2	47.0	-2.8	-4.8	-0.8
Tuberculosis: with HIV infection	45.8	46.5	-0.7	-2.6	1.1
Severe chest injury	47.6	51.2	-3.7	-5.6	-1.8
Iodine-deficiency goiter	51.3	51.1	0.2	-1.6	2.1
Burns: long-term	54.0	48.8	5.2	3.1	7.2
Mastectomy	52.6	55.2	-2.6	-4.5	-0.6
Herpes zoster	52.5	56.2	-3.7	-5.7	-1.7
Severe tooth loss	56.5	58.6	-2.1	-4.2	0.0
Impotence	68.1	69.0	-0.9	-2.9	1.1
Average effect of adding information, across all questions:					-0.8

Table 3 shows estimates of the impact of adding information (i.e., the coefficient β) for three different models. The crude model without any adjustments shows that, on average, adding social, psychological, or familial consequences to the health state description lowered individual ratings of that description by 0.78 points (95% confidence interval [CI]: -1.01 to -0.56). Adjusting for demographic covariates and indicator variable for each question had little impact on the crude estimate, thus for parsimony and transparency we did not adjust for covariates in the other analyses.

**Table 3:** Crude, demographic adjusted, and demographic and question-adjusted effect of adding information on health ratings.

	Crude		+Demographics		+Questions*	
	*β*	95% CI	*β*	95% CI	*β*	95% CI
**Added information**						
Yes vs. No	-0.78	[-1.01,-0.56]	-0.83	[-1.06,-0.60]	-0.83	[-1.06,-0.60]
**Age group**						
25-34y vs. <25y			1.93	[0.45,3.42]	1.93	[0.45,3.42]
35-44y vs. <25y			1.66	[-0.04,3.35]	1.66	[-0.04,3.35]
45+y vs. <25y			2.36	[0.32,4.40]	2.36	[0.32,4.40]
**Gender**						
Women vs. Men			-0.54	[-1.81,0.73]	-0.54	[-1.81,0.73]
**Race**						
Indian vs. White			-1.06	[-3.57,1.46]	-1.06	[-3.57,1.46]
Asian vs. White			-1.26	[-3.60,1.09]	-1.26	[-3.60,1.09]
Other vs. White			-2.89	[-4.87,-0.90]	-2.89	[-4.88,-0.90]
**Education**						
University vs. ≤HS			0.75	[-0.54,2.05]	0.75	[-0.54,2.05]
Graduate+ vs. ≤HS			1.56	[-0.18,3.30]	1.56	[-0.18,3.30]
**Survey time**						
10–19 vs. <10 mins			1.65	[0.27,3.02]	1.65	[0.27,3.03]
20+ vs. <10 mins			4.43	[2.60,6.27]	4.43	[2.60,6.27]
*N*	57312		54720		54720	

*Coefficients for individual questions omitted. 95% CI (clustered by respondent) in brackets.

**Fig 2:**
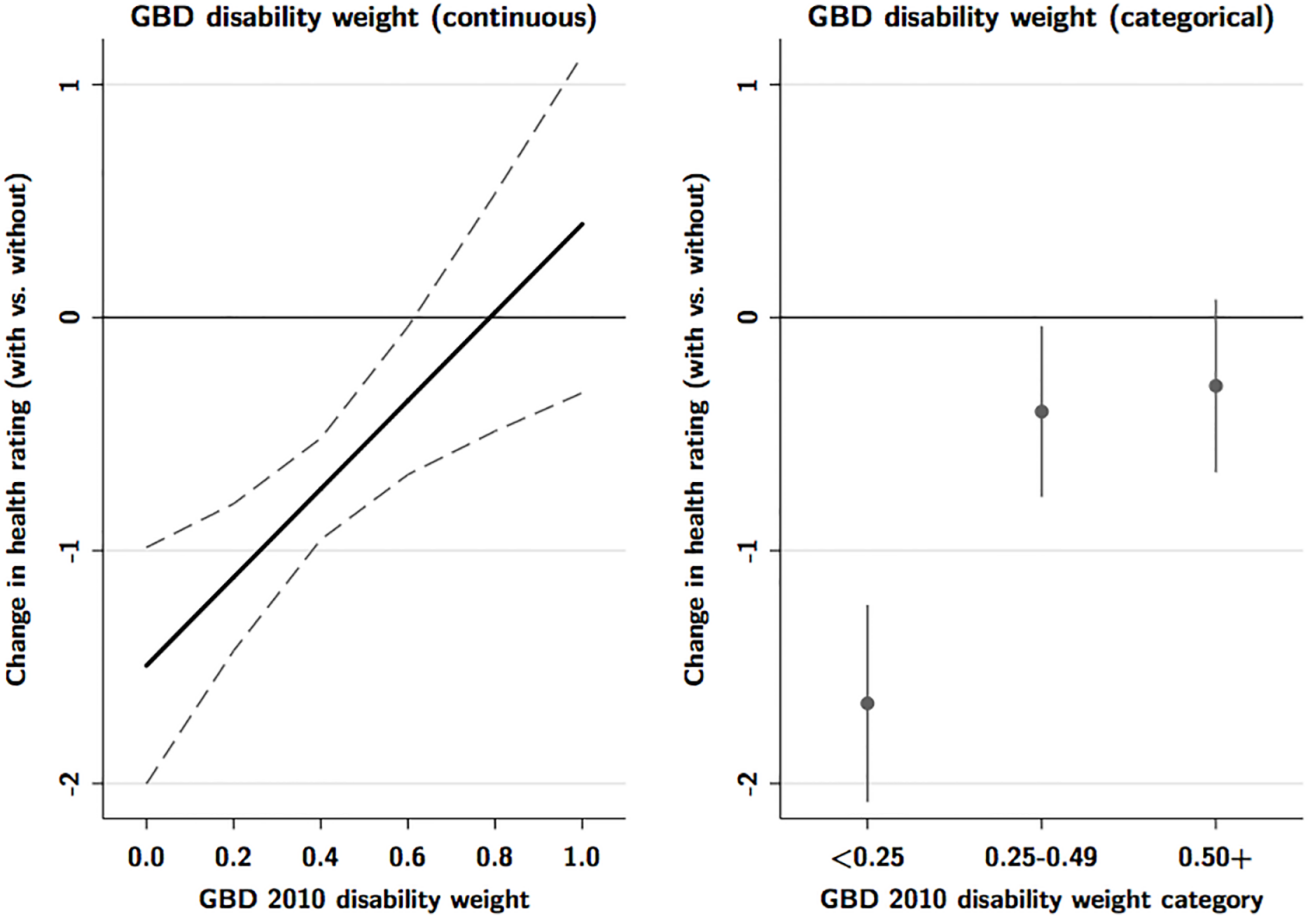
Differential effect of additional information by Global Burden of Disease disability weight (continuous and categorical).

To test hypothesis (2), we allowed the effect of added information to vary according to GBD disability weight. Fig 2 shows the marginal effects from models that included product terms between the main treatment variable and GBD score. When looked at as a continuous variable, the impact of adding information is strongest for health states with the lowest GBD disability weight (least severe conditions), and declines as GBD disability weight increases. However, this assumes that GBD disability weight has a linear relationship with ratings of health state descriptions. We also categorized the GBD disability weight into broad categories of mild (<-0.25), moderate (0.25–0.49), and severe (0.50+). When looked at categorically, adding information reduced disease ratings by 1.66 points (95% CI 1.2 to 2.1), for the least severe conditions (GBD weights <0.25), but showed weaker effects at higher GBD weight categories.

**Fig 3:**
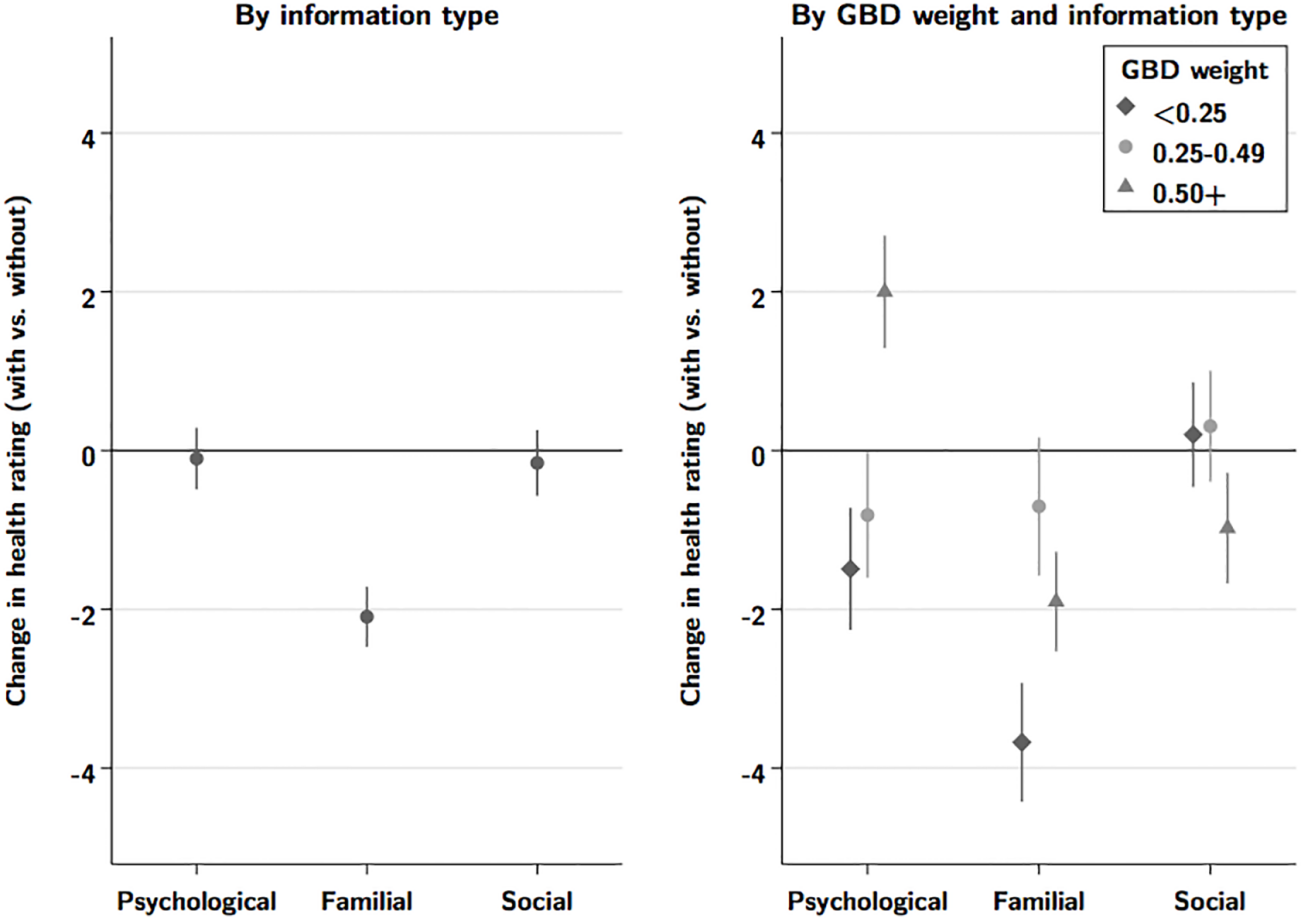
Question-specific effect of added information, by category of added information.

Fig 3 shows results from analyses allowing the impact of adding information to vary by the type of information added (psychological, social, or familial). Overall, adding information about familial consequences showed the greatest impact, reducing ratings by an average of 2.09 points (95%CI 1.7 to 2.5), compared to reductions of 0.10 for psychological and 0.16 for social implications. The different types of information also showed some evidence of heterogeneity by GBD category, with only familial information showing a consistently negative impact for all disability weight categories. Finally, we found limited evidence that the effect of adding information varied by gender, education, or age.

## Discussion

We hypothesized that the inclusion of psychological, social, or familial consequences related to health state descriptions will reduce the average rating of health, possibly due to an “unpacking effect” or other psychological phenomenon. We found that inclusion of information that is not directly related to major functional consequences or symptoms reduced average health ratings by 0.78 on a scale of 100. There was considerable heterogeneity within our sample: adding non-health information varied from reducing health rating by 9.1 points (traumatic brain injury) to increasing it by 5.19 points (end-stage renal disease). While this these differences did not consistently support our hypothesis that additional information would reduce average ratings, it is possible that—as found in other research on unpacking effects—added detail can produce more extreme evaluative judgments in either direction.[8]

We also hypothesized that the effect of including additional information would vary by disability weight (i.e. stronger for health conditions with lower disability weights), type of information (i.e. psychological, social, or familial consequences), and gender (i.e. the impact of adding information on familial will be stronger for women than men), but would not vary by education level or age.

We found some confirmatory evidence that adding information had a stronger impact on ratings of the least severe conditions, reducing average ratings in this category by 1.67 points, and that addition of information about child-rearing had a stronger impact than psychological or social consequences, reducing average ratings by 2.09 points. We found little evidence that the effect of adding information on ratings of health descriptions varied by gender, education, or age.

Overall, we found that inclusion of non-health information in health state descriptions could impact individuals’ evaluations of the severity of those health states. However, this impact was not consistent across all conditions or types of information, and was most pronounced for inclusion of information about child-rearing, and among the least severe conditions.

Our study has limitations. Use of a web-based survey rather than in-person testing methods may have impacted our results, and thus limit the generalizability of our findings. Our sample was drawn exclusively from literate individuals with computer and internet access, so generalizability is limited. It is unknown whether the effects that we discovered are large or consistent enough to impact actual relative rankings of conditions in a study such as the Global Burden of Disease; nor whether this effect would be present when using paired comparisons (as the GBD does) rather than a visual analogue scale.

## Conclusion

We found some evidence that inclusion of information about health states not directly related to major functional consequences or symptoms, particularly with respect to child-rearing and specifically for descriptions of less severe conditions, can lead to lower ratings of health, although the effect size was small and this finding was not consistent across health states. Future studies that attempt to isolate evaluations of ‘health’ should be consistent in their inclusion or exclusion of this type of information in order to facilitate the interpretation of subsequent findings, and ensure appropriate comparability of health states.

## Supporting information

S1 Text Survey versions 1 and 2. (PDF)

S1 Table Location of ISP addresses of respondents. (DOCX)
