# Supplementary material for: The impact of social and psychological consequences of disease on judgments of disease severity: An experimental study
Source: PLoS One. 2018 Apr 17;13(4):e0195338. doi: 10.1371/journal.pone.0195338 (PMC5903632; doi:10.1371/journal.pone.0195338)
Supplement: S1 Text — (PDF) [file pone.0195338.s001.pdf]

Survey Version 1

A Survey of Health Ratings

Page #1

(Introduction and consent statement.)

I have read the above statement and agree to participate in this survey.

Yes, I understand and agree.

Ratings

The following questions will present you with a set of symptoms associated with a health condition. Read each description and assign a value between 0 and 100 to it that shows how healthy or unhealthy you think the person with that health condition is. (Where 0 indicates a person with the worst possible health, and 100 indicates a person with the best possible health.)

1) Please consider the following health condition:

The person had a severe chest injury in the past that has now healed. The person still gets breathless when walking, feels discomfort in the chest, and is sometimes angry and depressed.

Please assign a value between 0 and 100 that shows how healthy or unhealthy you think a person with that health condition is. 0 indicates a person with the worst possible health, and 100 indicates a person with the best possible health.

____

2) Please consider the following health condition:

The person cannot move around without help, and cannot lift or hold objects, get dressed or sit upright. The person also has low intelligence, speaks few words, and needs a lot of help with all basic daily activities. The person cannot raise children.

Please assign a value between 0 and 100 that shows how healthy or unhealthy you think a person with that health condition is. 0 indicates a person with the worst possible health, and 100 indicates a person with the best possible health.

____

3) Please consider the following health condition:

The person is tired, is sometimes angry and depressed, and has itching, cramps, headache, joint pains and shortness of breath. The person needs intensive medical care every other day lasting about half a day.

Please assign a value between 0 and 100 that shows how healthy or unhealthy you think a person with that health condition is. 0 indicates a person with the worst possible health, and 100 indicates a person with the best possible health.

____

4) Please consider the following health condition:

The person is confined to bed or a wheelchair, depends on others for feeding, toileting and dressing, and has difficulty thinking clearly and remembering things.

Please assign a value between 0 and 100 that shows how healthy or unhealthy you think a person with that health condition is. 0 indicates a person with the worst possible health, and 100 indicates a person with the best possible health.

____

5) Please consider the following health condition:

The person is paralyzed from the neck down and cannot feel or move the arms and legs.

Please assign a value between 0 and 100 that shows how healthy or unhealthy you think a person with that health condition is. 0 indicates a person with the worst possible health, and 100 indicates a person with the best possible health.

____

6) Please consider the following health condition:

The person has frequent headaches, memory problems, difficulty concentrating, and dizziness. The person needs help to raise children.

Please assign a value between 0 and 100 that shows how healthy or unhealthy you think a person with that health condition is. 0 indicates a person with the worst possible health, and 100 indicates a person with the best possible health.

____

7) Please consider the following health condition:

The person has scars caused by burns over a large part of the body. The scars are frequently painful and itchy.

Please assign a value between 0 and 100 that shows how healthy or unhealthy you think a person with that health condition is. 0 indicates a person with the worst possible health, and 100 indicates a person with the best possible health.

____

8) Please consider the following health condition:

The person is confined to bed or a wheelchair, has difficulty communicating with others, and depends on others for feeding, toileting and dressing.

Please assign a value between 0 and 100 that shows how healthy or unhealthy you think a person with that health condition is. 0 indicates a person with the worst possible health, and 100 indicates a person with the best possible health.

____

9) Please consider the following health condition:

The person is hyperactive, hears and believes things that are not real, and engages in impulsive and aggressive behaviour.

Please assign a value between 0 and 100 that shows how healthy or unhealthy you think a person with that health condition is. 0 indicates a person with the worst possible health, and 100 indicates a person with the best possible health.

____

10) Please consider the following health condition:

The person has a pouch attached to an opening in the belly to collect and empty stools.

Please assign a value between 0 and 100 that shows how healthy or unhealthy you think a person with that health condition is. 0 indicates a person with the worst possible health, and 100 indicates a person with the best possible health.

____

11) Please consider the following health condition:

The person had one of her breasts removed, which is obvious to others. The person sometimes has pain or swelling in the arms.

Please assign a value between 0 and 100 that shows how healthy or unhealthy you think a person with that health condition is. 0 indicates a person with the worst possible health, and 100 indicates a person with the best possible health.

____

12) Please consider the following health condition:

The person needs help walking, has difficulty with writing and arm coordination, has loss of vision in one eye and cannot control urinating. The person is sometimes angry and depressed.

Please assign a value between 0 and 100 that shows how healthy or unhealthy you think a person with that health condition is. 0 indicates a person with the worst possible health, and 100 indicates a person with the best possible health.

____

13) Please consider the following health condition:

The person is paralyzed from the neck down and cannot feel or move the arms and legs. Arms and legs are in fixed, bent positions, and the person gets frequent infections and pressure sores. The person requires a lot of support to raise children.

Please assign a value between 0 and 100 that shows how healthy or unhealthy you think a person with that health condition is. 0 indicates a person with the worst possible health, and 100 indicates a person with the best possible health.

____

14) Please consider the following health condition:

The person has low intelligence and requires constant assistance for nearly all activities.

Please assign a value between 0 and 100 that shows how healthy or unhealthy you think a person with that health condition is. 0 indicates a person with the worst possible health, and 100 indicates a person with the best possible health.

____

15) Please consider the following health condition:

The person is not breathing and has no pulse. The person is dead.

Please assign a value between 0 and 100 that shows how healthy or unhealthy you think a person with that health condition is. 0 indicates a person with the worst possible health, and 100 indicates a person with the best possible health.

____

16) Please consider the following health condition:

The person has lost a lot of weight and regularly uses strong medication to avoid constant pain. The person has no appetite, feels nauseous, and needs to spend most of the day in bed.

Please assign a value between 0 and 100 that shows how healthy or unhealthy you think a person with that health condition is. 0 indicates a person with the worst possible health, and 100 indicates a person with the best possible health.

____

17) Please consider the following health condition:

The person has severe tremors and moves very slowly, which causes great difficulty in walking and daily activities. The person falls easily and has difficulty communicating with others, swallowing, sleeping, and remembering things.

Please assign a value between 0 and 100 that shows how healthy or unhealthy you think a person with that health condition is. 0 indicates a person with the worst possible health, and 100 indicates a person with the best possible health.

____

18) Please consider the following health condition:

The person has an abnormal opening between her vagina and rectum causing flatulence and feces to escape through the vagina, and sometimes causing significant others to notice and comment. The person gets infections in her vagina, and has pain when urinating.

Please assign a value between 0 and 100 that shows how healthy or unhealthy you think a person with that health condition is. 0 indicates a person with the worst possible health, and 100 indicates a person with the best possible health.

____

19) Please consider the following health condition:

The person has a painful burn over a large part of the body. Parts of the burned area have lost feeling, and the person feels unwell.

Please assign a value between 0 and 100 that shows how healthy or unhealthy you think a person with that health condition is. 0 indicates a person with the worst possible health, and 100 indicates a person with the best possible health.

____

20) Please consider the following health condition:

The person has some difficulty in moving around, holding objects, dressing and sitting upright, and is slow in learning to speak and do simple tasks. The person can walk without help, but requires a lot of help with daily activities and raising children.

Please assign a value between 0 and 100 that shows how healthy or unhealthy you think a person with that health condition is. 0 indicates a person with the worst possible health, and 100 indicates a person with the best possible health.

____

21) Please consider the following health condition:

Set the rating for this person at "78".

Please assign a value between 0 and 100 that shows how healthy or unhealthy you think a person with that health condition is. 0 indicates a person with the worst possible health, and 100 indicates a person with the best possible health.

____

22) Please consider the following health condition:

The person has slurred speech and difficulty swallowing. The person has weak arms and hands, very limited and stiff leg movement, has loss of vision in both eyes and cannot control urinating.

Please assign a value between 0 and 100 that shows how healthy or unhealthy you think a person with that health condition is. 0 indicates a person with the worst possible health, and 100 indicates a person with the best possible health.

____

23) Please consider the following health condition:

The person has a persistent cough and fever, shortness of breath, night sweats, weakness and fatigue, severe weight loss, and is sometimes angry and depressed.

Please assign a value between 0 and 100 that shows how healthy or unhealthy you think a person with that health condition is. 0 indicates a person with the worst possible health, and 100 indicates a person with the best possible health.

____

24) Please consider the following health condition:

The person has a large mass in the front of the neck that causes others to stare and comment. The person sometimes has weakness and fatigue, constipation and weight gain.

Please assign a value between 0 and 100 that shows how healthy or unhealthy you think a person with that health condition is. 0 indicates a person with the worst possible health, and 100 indicates a person with the best possible health.

____

25) Please consider the following health condition:

The person uses heroin daily and has difficulty controlling the habit. When the effects wear off, the person feels severe nausea, agitation, vomiting and fever, and is sometimes angry and depressed. The person has a lot of difficulty in daily activities.

Please assign a value between 0 and 100 that shows how healthy or unhealthy you think a person with that health condition is. 0 indicates a person with the worst possible health, and 100 indicates a person with the best possible health.

____

26) Please consider the following health condition:

Set the rating for this person at "44".

Please assign a value between 0 and 100 that shows how healthy or unhealthy you think a person with that health condition is. 0 indicates a person with the worst possible health, and 100 indicates a person with the best possible health.

____

27) Please consider the following health condition:

The person hears and sees things that are not real. The person can be forgetful and has difficulty with daily activities. The person often needs help to raise children.

Please assign a value between 0 and 100 that shows how healthy or unhealthy you think a person with that health condition is. 0 indicates a person with the worst possible health, and 100 indicates a person with the best possible health.

____

28) Please consider the following health condition:

The person has lost more than 20 teeth including front and back, and has great difficulty in eating meat, fruits, and vegetables.

Please assign a value between 0 and 100 that shows how healthy or unhealthy you think a person with that health condition is. 0 indicates a person with the worst possible health, and 100 indicates a person with the best possible health.

____

29) Please consider the following health condition:

The person has complete memory loss; no longer recognizes close family members; and requires help with all daily activities. The person cannot raise children.

Please assign a value between 0 and 100 that shows how healthy or unhealthy you think a person with that health condition is. 0 indicates a person with the worst possible health, and 100 indicates a person with the best possible health.

____

30) Please consider the following health condition:

The person has diarrhea three or more times a day with severe belly cramps. The person is very thirsty and feels nauseous and tired.

Please assign a value between 0 and 100 that shows how healthy or unhealthy you think a person with that health condition is. 0 indicates a person with the worst possible health, and 100 indicates a person with the best possible health.

____

31) Please consider the following health condition:

The person has a blistering skin rash that causes pain with some burning and itching and is sometimes angry and depressed.

Please assign a value between 0 and 100 that shows how healthy or unhealthy you think a person with that health condition is. 0 indicates a person with the worst possible health, and 100 indicates a person with the best possible health.

____

32) Please consider the following health condition:

The person has severe, constant pain and deformity in most joints, causing difficulty moving around, getting up and down, eating, dressing, lifting, carrying and using the hands. The person often feels extreme fatigue.

Please assign a value between 0 and 100 that shows how healthy or unhealthy you think a person with that health condition is. 0 indicates a person with the worst possible health, and 100 indicates a person with the best possible health.

____

33) Please consider the following health condition:

The person has no aches or pains, has no difficulties and feels perfectly well.

Please assign a value between 0 and 100 that shows how healthy or unhealthy you think a person with that health condition is. 0 indicates a person with the worst possible health, and 100 indicates a person with the best possible health.

____

34) Please consider the following health condition:

The person cannot think clearly and has frequent headaches, memory problems, difficulty concentrating and dizziness. The person depends on others for feeding, toileting, dressing and walking.

Please assign a value between 0 and 100 that shows how healthy or unhealthy you think a person with that health condition is. 0 indicates a person with the worst possible health, and 100 indicates a person with the best possible health.

____

35) Please consider the following health condition:

The person is paralyzed from the waist down and cannot feel or move the legs. Legs are in fixed, bent positions, and the person gets frequent infections and pressure sores.

Please assign a value between 0 and 100 that shows how healthy or unhealthy you think a person with that health condition is. 0 indicates a person with the worst possible health, and 100 indicates a person with the best possible health.

____

36) Please consider the following health condition:

The person has pain and deformity in most joints, causing difficulty moving around, getting up and down, and using the hands for lifting and carrying. The person often feels fatigue.

Please assign a value between 0 and 100 that shows how healthy or unhealthy you think a person with that health condition is. 0 indicates a person with the worst possible health, and 100 indicates a person with the best possible health.

____

37) Please consider the following health condition:

The person has severe vision loss, which causes difficulty in daily activities and some difficulty going outside the home without assistance.

Please assign a value between 0 and 100 that shows how healthy or unhealthy you think a person with that health condition is. 0 indicates a person with the worst possible health, and 100 indicates a person with the best possible health.

____

38) Please consider the following health condition:

The person has difficulty in obtaining or maintaining an erection.

Please assign a value between 0 and 100 that shows how healthy or unhealthy you think a person with that health condition is. 0 indicates a person with the worst possible health, and 100 indicates a person with the best possible health.

____

39) Please consider the following health condition:

The person has an abnormal opening between the bladder and the vagina, which makes her unable to control urinating, and may cause significant others to notice and comment.

Please assign a value between 0 and 100 that shows how healthy or unhealthy you think a person with that health condition is. 0 indicates a person with the worst possible health, and 100 indicates a person with the best possible health.

____

40) Please consider the following health condition:

The person has severe weight loss, weakness, fatigue, cough and fever, and frequent infections, skin rashes and diarrhea.

Please assign a value between 0 and 100 that shows how healthy or unhealthy you think a person with that health condition is. 0 indicates a person with the worst possible health, and 100 indicates a person with the best possible health.

____

Survey Version 2

Page #1

(Introduction and consent statement.)

I have read the above statement and agree to participate in this survey.

Yes, I understand and agree.

Ratings

The following questions will present you with a set of symptoms associated with a health condition. Read each description and assign a value between 0 and 100 to it that shows how healthy or unhealthy you think the person with that health condition is. (Where 0 indicates a person with the worst possible health, and 100 indicates a person with the best possible health.)

1) Please consider the following health condition:

The person had a severe chest injury in the past that has now healed. The person still gets breathless when walking and feels discomfort in the chest.

Please assign a value between 0 and 100 that shows how healthy or unhealthy you think a person with that health condition is. 0 indicates a person with the worst possible health, and 100 indicates a person with the best possible health.

____

2) Please consider the following health condition:

The person cannot move around without help, and cannot lift or hold objects, get dressed or sit upright. The person also has low intelligence, speaks few words, and needs a lot of help with all basic daily activities.

Please assign a value between 0 and 100 that shows how healthy or unhealthy you think a person with that health condition is. 0 indicates a person with the worst possible health, and 100 indicates a person with the best possible health.

____

3) Please consider the following health condition:

The person is tired and has itching, cramps, headache, joint pains and shortness of breath. The person needs intensive medical care every other day lasting about half a day.

Please assign a value between 0 and 100 that shows how healthy or unhealthy you think a person with that health condition is. 0 indicates a person with the worst possible health, and 100 indicates a person with the best possible health.

____

4) Please consider the following health condition:

The person is confined to bed or a wheelchair, depends on others for feeding, toileting and dressing, and has difficulty thinking clearly and remembering things. The person requires a lot of support to raise children.

Please assign a value between 0 and 100 that shows how healthy or unhealthy you think a person with that health condition is. 0 indicates a person with the worst possible health, and 100 indicates a person with the best possible health.

____

5) Please consider the following health condition:

The person is paralyzed from the neck down and cannot feel or move the arms and legs. The person is sometimes angry and depressed.

Please assign a value between 0 and 100 that shows how healthy or unhealthy you think a person with that health condition is. 0 indicates a person with the worst possible health, and 100 indicates a person with the best possible health.

____

6) Please consider the following health condition:

The person has frequent headaches, memory problems, difficulty concentrating, and dizziness.

Please assign a value between 0 and 100 that shows how healthy or unhealthy you think a person with that health condition is. 0 indicates a person with the worst possible health, and 100 indicates a person with the best possible health.

____

7) Please consider the following health condition:

The person has scars caused by burns over a large part of the body, which cause others to stare and comment. The scars are frequently painful and itchy.

Please assign a value between 0 and 100 that shows how healthy or unhealthy you think a person with that health condition is. 0 indicates a person with the worst possible health, and 100 indicates a person with the best possible health.

____

8) Please consider the following health condition:

The person is confined to bed or a wheelchair and depends on others for feeding, toileting and dressing.

Please assign a value between 0 and 100 that shows how healthy or unhealthy you think a person with that health condition is. 0 indicates a person with the worst possible health, and 100 indicates a person with the best possible health.

____

9) Please consider the following health condition:

The person is hyperactive, hears and believes things that are not real, and engages in impulsive and aggressive behavior. The person requires a lot of support to raise children.

Please assign a value between 0 and 100 that shows how healthy or unhealthy you think a person with that health condition is. 0 indicates a person with the worst possible health, and 100 indicates a person with the best possible health.

____

10) Please consider the following health condition:

The person has a pouch attached to an opening in the belly to collect and empty stools and is sometimes angry and depressed.

Please assign a value between 0 and 100 that shows how healthy or unhealthy you think a person with that health condition is. 0 indicates a person with the worst possible health, and 100 indicates a person with the best possible health.

____

11) Please consider the following health condition:

The person had one of her breasts removed and sometimes has pain or swelling in the arms.

Please assign a value between 0 and 100 that shows how healthy or unhealthy you think a person with that health condition is. 0 indicates a person with the worst possible health, and 100 indicates a person with the best possible health.

____

12) Please consider the following health condition:

The person needs help walking, has difficulty with writing and arm coordination, has loss of vision in one eye and cannot control urinating.

Please assign a value between 0 and 100 that shows how healthy or unhealthy you think a person with that health condition is. 0 indicates a person with the worst possible health, and 100 indicates a person with the best possible health.

____

13) Please consider the following health condition:

The person is paralyzed from the neck down and cannot feel or move the arms and legs. Arms and legs are in fixed, bent positions, and the person gets frequent infections and pressure sores.

Please assign a value between 0 and 100 that shows how healthy or unhealthy you think a person with that health condition is. 0 indicates a person with the worst possible health, and 100 indicates a person with the best possible health.

____

14) Please consider the following health condition:

The person has low intelligence and requires constant assistance for nearly all activities. As an adult, the person cannot raise children.

Please assign a value between 0 and 100 that shows how healthy or unhealthy you think a person with that health condition is. 0 indicates a person with the worst possible health, and 100 indicates a person with the best possible health.

____

15) Please consider the following health condition:

The person is not breathing and has no pulse. The person is dead.

Please assign a value between 0 and 100 that shows how healthy or unhealthy you think a person with that health condition is. 0 indicates a person with the worst possible health, and 100 indicates a person with the best possible health.

____

16) Please consider the following health condition:

The person has lost a lot of weight and regularly uses strong medication to avoid constant pain. The person is sometimes angry and depressed, has no appetite, feels nauseous, and needs to spend most of the day in bed.

Please assign a value between 0 and 100 that shows how healthy or unhealthy you think a person with that health condition is. 0 indicates a person with the worst possible health, and 100 indicates a person with the best possible health.

____

17) Please consider the following health condition:

The person has severe tremors and moves very slowly, which causes great difficulty in walking and daily activities. The person falls easily and has a lot of difficulty swallowing, sleeping, and remembering things.

Please assign a value between 0 and 100 that shows how healthy or unhealthy you think a person with that health condition is. 0 indicates a person with the worst possible health, and 100 indicates a person with the best possible health.

____

18) Please consider the following health condition:

The person has an abnormal opening between her vagina and rectum causing flatulence and feces to escape through the vagina. The person gets infections in her vagina, and has pain when urinating.

Please assign a value between 0 and 100 that shows how healthy or unhealthy you think a person with that health condition is. 0 indicates a person with the worst possible health, and 100 indicates a person with the best possible health.

____

19) Please consider the following health condition:

The person has a painful burn over a large part of the body that causes others notice. Parts of the burned area have lost feeling, and the person feels unwell.

Please assign a value between 0 and 100 that shows how healthy or unhealthy you think a person with that health condition is. 0 indicates a person with the worst possible health, and 100 indicates a person with the best possible health.

____

20) Please consider the following health condition:

The person has some difficulty in moving around, holding objects, dressing and sitting upright, and is slow in learning to speak and do simple tasks. The person can walk without help, but requires a lot of help with daily activities.

Please assign a value between 0 and 100 that shows how healthy or unhealthy you think a person with that health condition is. 0 indicates a person with the worst possible health, and 100 indicates a person with the best possible health.

____

21) Please consider the following health condition:

Set the rating for this person at "78".

Please assign a value between 0 and 100 that shows how healthy or unhealthy you think a person with that health condition is. 0 indicates a person with the worst possible health, and 100 indicates a person with the best possible health.

____

22) Please consider the following health condition:

The person has slurred speech and difficulty swallowing and has difficulty communicating with others. The person has weak arms and hands, very limited and stiff leg movement, has loss of vision in both eyes and cannot control urinating.

Please assign a value between 0 and 100 that shows how healthy or unhealthy you think a person with that health condition is. 0 indicates a person with the worst possible health, and 100 indicates a person with the best possible health.

____

23) Please consider the following health condition:

The person has a persistent cough and fever, shortness of breath, night sweats, weakness and fatigue and severe weight loss.

Please assign a value between 0 and 100 that shows how healthy or unhealthy you think a person with that health condition is. 0 indicates a person with the worst possible health, and 100 indicates a person with the best possible health.

24) Please consider the following health condition:

The person has a large mass in the front of the neck. The person sometimes has weakness and fatigue, constipation and weight gain.

Please assign a value between 0 and 100 that shows how healthy or unhealthy you think a person with that health condition is. 0 indicates a person with the worst possible health, and 100 indicates a person with the best possible health.

25) Please consider the following health condition:

The person uses heroin daily and has difficulty controlling the habit. When the effects wear off, the person feels severe nausea, agitation, vomiting and fever. The person has a lot of difficulty in daily activities.

Please assign a value between 0 and 100 that shows how healthy or unhealthy you think a person with that health condition is. 0 indicates a person with the worst possible health, and 100 indicates a person with the best possible health.

____

26) Please consider the following health condition:

Set the rating for this person at "44".

Please assign a value between 0 and 100 that shows how healthy or unhealthy you think a person with that health condition is. 0 indicates a person with the worst possible health, and 100 indicates a person with the best possible health.

____

27) Please consider the following health condition:

The person hears and sees things that are not real. The person can be forgetful and has difficulty with daily activities.

Please assign a value between 0 and 100 that shows how healthy or unhealthy you think a person with that health condition is. 0 indicates a person with the worst possible health, and 100 indicates a person with the best possible health.

____

28) Please consider the following health condition:

The person has lost more than 20 teeth including front and back, has great difficulty in eating meat, fruits, and vegetables, and has difficulty communicating with others.

Please assign a value between 0 and 100 that shows how healthy or unhealthy you think a person with that health condition is. 0 indicates a person with the worst possible health, and 100 indicates a person with the best possible health.

____

29) Please consider the following health condition:

The person has complete memory loss; no longer recognizes close family members; and requires help with all daily activities.

Please assign a value between 0 and 100 that shows how healthy or unhealthy you think a person with that health condition is. 0 indicates a person with the worst possible health, and 100 indicates a person with the best possible health.

____

30) Please consider the following health condition:

The person has diarrhea three or more times a day with severe belly cramps. The person is very thirsty and feels nauseous, tired, and is sometimes angry and depressed.

Please assign a value between 0 and 100 that shows how healthy or unhealthy you think a person with that health condition is. 0 indicates a person with the worst possible health, and 100 indicates a person with the best possible health.

____

31) Please consider the following health condition:

The person has a blistering skin rash that causes pain, with some burning and itching.

Please assign a value between 0 and 100 that shows how healthy or unhealthy you think a person with that health condition is. 0 indicates a person with the worst possible health, and 100 indicates a person with the best possible health.

32) Please consider the following health condition:

The person has severe, constant pain and deformity in most joints that others notice. The person has difficulty moving around, getting up and down, eating, dressing, lifting, carrying and using the hands. The person often feels extreme fatigue.

Please assign a value between 0 and 100 that shows how healthy or unhealthy you think a person with that health condition is. 0 indicates a person with the worst possible health, and 100 indicates a person with the best possible health.

33) Please consider the following health condition:

The person has no aches or pains, has no difficulties and feels perfectly well.

Please assign a value between 0 and 100 that shows how healthy or unhealthy you think a person with that health condition is. 0 indicates a person with the worst possible health, and 100 indicates a person with the best possible health.

____

34) Please consider the following health condition:

The person cannot think clearly and has frequent headaches, memory problems, difficulty concentrating and dizziness. The person depends on others for feeding, toileting, dressing and walking. The person requires a lot of support to raise children.

Please assign a value between 0 and 100 that shows how healthy or unhealthy you think a person with that health condition is. 0 indicates a person with the worst possible health, and 100 indicates a person with the best possible health.

____

35) Please consider the following health condition:

The person is paralyzed from the waist down and cannot feel or move the legs. Legs are in fixed, bent positions, and the person gets frequent infections and pressure sores. The person requires a lot of support to raise children.

Please assign a value between 0 and 100 that shows how healthy or unhealthy you think a person with that health condition is. 0 indicates a person with the worst possible health, and 100 indicates a person with the best possible health.

____

36) Please consider the following health condition:

The person has pain and deformity in most joints that others notice. The person has difficulty moving around, getting up and down, and using the hands for lifting and carrying. The person often feels fatigue.

Please assign a value between 0 and 100 that shows how healthy or unhealthy you think a person with that health condition is. 0 indicates a person with the worst possible health, and 100 indicates a person with the best possible health.

____

37) Please consider the following health condition:

The person has severe vision loss, which causes difficulty in daily activities and some difficulty going outside the home without assistance. The person often needs help to raise children.

Please assign a value between 0 and 100 that shows how healthy or unhealthy you think a person with that health condition is. 0 indicates a person with the worst possible health, and 100 indicates a person with the best possible health.

____

38) Please consider the following health condition:

The person has difficulty in obtaining or maintaining an erection and is sometimes angry and depressed.

Please assign a value between 0 and 100 that shows how healthy or unhealthy you think a person with that health condition is. 0 indicates a person with the worst possible health, and 100 indicates a person with the best possible health.

____

39) Please consider the following health condition:

The person has an abnormal opening between the bladder and the vagina, which makes her unable to control urinating.

Please assign a value between 0 and 100 that shows how healthy or unhealthy you think a person with that health condition is. 0 indicates a person with the worst possible health, and 100 indicates a person with the best possible health.

40) Please consider the following health condition:

The person has severe weight loss, weakness, fatigue, cough and fever, and frequent infections, skin rashes and diarrhea, and is sometimes angry and depressed.

Please assign a value between 0 and 100 that shows how healthy or unhealthy you think a person with that health condition is. 0 indicates a person with the worst possible health, and 100 indicates a person with the best possible health.
