# Supplementary material for: The impact of social and psychological consequences of disease on judgments of disease severity: An experimental study
Source: PLoS One. 2018 Apr 17;13(4):e0195338. doi: 10.1371/journal.pone.0195338 (PMC5903632; doi:10.1371/journal.pone.0195338)
Supplement: S1 Table — (DOCX) [file pone.0195338.s002.docx]

                location |      Freq.     Percent        Cum.

--------------------------+-----------------------------------

                    India |        138        8.87        8.87

                    Spain |         79        5.08       13.95

   Bosnia and Herzegovina |         77        4.95       18.89

                   Serbia |         75        4.82       23.71

                  Romania |         73        4.69       28.41

           United Kingdom |         72        4.63       33.03

                    Italy |         67        4.31       37.34

                 Bulgaria |         54        3.47       40.81

                Venezuela |         46        2.96       43.77

                 Portugal |         45        2.89       46.66

       Russian Federation |         45        2.89       49.55

                   Poland |         38        2.44       51.99

                Argentina |         33        2.12       54.11

                   Greece |         30        1.93       56.04

                Indonesia |         30        1.93       57.97

                  Croatia |         29        1.86       59.83

                   Turkey |         27        1.74       61.57

                 Pakistan |         26        1.67       63.24

                   Mexico |         24        1.54       64.78

                  Germany |         23        1.48       66.26

                  Hungary |         22        1.41       67.67

              Philippines |         21        1.35       69.02

                   Brazil |         20        1.29       70.31

                  Vietnam |         20        1.29       71.59

              Netherlands |         19        1.22       72.81

                  Ontario |         19        1.22       74.04

                Macedonia |         17        1.09       75.13

                  Ukraine |         17        1.09       76.22

                  Morocco |         14        0.90       77.12

                   Sweden |         13        0.84       77.96

                 New York |         12        0.77       78.73

                 Slovenia |         12        0.77       79.50

               Bangladesh |         11        0.71       80.21

                  Finland |         11        0.71       80.91

                   France |         11        0.71       81.62

                  Austria |         10        0.64       82.26

                 Colombia |         10        0.64       82.90

                 Malaysia |         10        0.64       83.55

                 Slovakia |         10        0.64       84.19

                  Belgium |          9        0.58       84.77

                    Egypt |          9        0.58       85.35

                  Ireland |          7        0.45       85.80

                Sri Lanka |          7        0.45       86.25

                Wisconsin |          7        0.45       86.70

                  Algeria |          6        0.39       87.08

                    Chile |          6        0.39       87.47

                  Estonia |          6        0.39       87.85

                  Georgia |          6        0.39       88.24

           Czech Republic |          5        0.32       88.56

       Dominican Republic |          5        0.32       88.88

              El Salvador |          5        0.32       89.20

                 Illinois |          5        0.32       89.52

                Lithuania |          5        0.32       89.85

           South Carolina |          5        0.32       90.17

                  Arizona |          4        0.26       90.42

         British Columbia |          4        0.26       90.68

               California |          4        0.26       90.94

                 Delaware |          4        0.26       91.20

                  Florida |          4        0.26       91.45

                Hong Kong |          4        0.26       91.71

                   Latvia |          4        0.26       91.97

                 Michigan |          4        0.26       92.22

                     Ohio |          4        0.26       92.48

             Pennsylvania |          4        0.26       92.74

                Singapore |          4        0.26       92.99

                Tennessee |          4        0.26       93.25

                 Thailand |          4        0.26       93.51

                  Uruguay |          4        0.26       93.77

                 Virginia |          4        0.26       94.02

                  Denmark |          3        0.19       94.22

                  Ecuador |          3        0.19       94.41

Newfoundland and Labrador |          3        0.19       94.60

                 Oklahoma |          3        0.19       94.79

             South Africa |          3        0.19       94.99

                    Texas |          3        0.19       95.18

                  Tunisia |          3        0.19       95.37

                  Albania |          2        0.13       95.50

                  Alberta |          2        0.13       95.63

                Australia |          2        0.13       95.76

                  Bolivia |          2        0.13       95.89

                     Iowa |          2        0.13       96.02

                  Jamaica |          2        0.13       96.14

                   Kansas |          2        0.13       96.27

                    Korea |          2        0.13       96.40

                    Nepal |          2        0.13       96.53

            New Brunswick |          2        0.13       96.66

               New Jersey |          2        0.13       96.79

           North Carolina |          2        0.13       96.92

                     Peru |          2        0.13       97.04

              Puerto Rico |          2        0.13       97.17

                   Quebec |          2        0.13       97.30

                   Taiwan |          2        0.13       97.43

               Washington |          2        0.13       97.56

            West Virginia |          2        0.13       97.69

                  Armenia |          1        0.06       97.75

                    China |          1        0.06       97.81

              Connecticut |          1        0.06       97.88

                   Cyprus |          1        0.06       97.94

                   Europe |          1        0.06       98.01

                Guatemala |          1        0.06       98.07

                 Honduras |          1        0.06       98.14

                  Indiana |          1        0.06       98.20

                   Israel |          1        0.06       98.26

                    Japan |          1        0.06       98.33

                   Jordan |          1        0.06       98.39

                 Kentucky |          1        0.06       98.46

                    Kenya |          1        0.06       98.52

                    Maine |          1        0.06       98.59

                 Manitoba |          1        0.06       98.65

            Massachusetts |          1        0.06       98.71

              Mississippi |          1        0.06       98.78

                 Missouri |          1        0.06       98.84

                  Moldova |          1        0.06       98.91

               Montenegro |          1        0.06       98.97

                   Nevada |          1        0.06       99.04

            New Hampshire |          1        0.06       99.10

               New Mexico |          1        0.06       99.16

              New Zealand |          1        0.06       99.23

             North Dakota |          1        0.06       99.29

                   Norway |          1        0.06       99.36

              Nova Scotia |          1        0.06       99.42

                   Oregon |          1        0.06       99.49

                 Paraguay |          1        0.06       99.55

                    Qatar |          1        0.06       99.61

             Saudi Arabia |          1        0.06       99.68

              Switzerland |          1        0.06       99.74

      Trinidad and Tobago |          1        0.06       99.81

                   Uganda |          1        0.06       99.87

     United Arab Emirates |          1        0.06       99.94

                     Utah |          1        0.06      100.00

--------------------------+-----------------------------------

                    Total |      1,556      100.00
